# A Combination of Xyloglucan, Pea Protein and Chia Seed Ameliorates Intestinal Barrier Integrity and Mucosa Functionality in a Rat Model of Constipation-Predominant Irritable Bowel Syndrome

**DOI:** 10.3390/jcm11237073

**Published:** 2022-11-29

**Authors:** Alessia Filippone, Alessio Ardizzone, Valentina Bova, Marika Lanza, Giovanna Casili, Salvatore Cuzzocrea, Emanuela Esposito, Michela Campolo, Irene Paterniti

**Affiliations:** Department of Chemical, Biological, Pharmaceutical and Environmental Sciences, University of Messina, Viale Ferdinando Stagno D’ Alcontres, 31, 98166 Messina, Italy

**Keywords:** irritable bowel syndrome (IBS), constipation, natural compounds, intestinal barrier

## Abstract

Irritable Bowel Syndrome is a gastrointestinal disorder that affects the large intestine, which encompasses several symptoms including, but not limited to, abdominal pain, bloating and dysmotility. In particular, IBS associated with constipation (IBS-C) is characterized by hard and dry stools and inadequate evacuation and difficulty in defecation. Although several drugs ameliorate intestinal modifications and constipation-associated features, management of IBS is still a challenge. Natural compounds including Xyloglucan and pea protein (XP) and Chia seed powder (CS) are widely known to possess beneficial effects in counteracting several gastrointestinal disorders. Here, we aimed to assess the combined effects of XP and CS to treat constipation-related alterations in an IBS-C rat model. IBS-C was induced by gastric instillation of 2 mL of cold water (0–4 °C) for 14 days and Xiloglucan, Pea protein and Chia seeds (XP + CS) treatment was orally administered for 7 days. On day 22, colon tissues were collected for histological analysis. Our results showed that XP + CS administration attenuated constipation-related parameters by increasing body weight and food and water intake. Upon XP + CS treatment, from day 14 to 22, stool moisture content was restored to physiological level. Colonic tissues from IBS-C rats depicted a disruption of the organ architecture accompanied by edema. Loss of colonic structure was reflected by the marked reduction of tight junction protein expression, Occludin and zona occludens-1 (ZO-1). Administration of XP + CS treatment in IBS-C rats significantly ameliorated the colonic histological parameters and exerted a positive effect on barrier integrity by restoring the expression of Occludin and zona occludens-1 (ZO-1). Our findings demonstrated that the efficacy of XP and CS in managing constipation in rats is due to the ability of these compounds to form a protective barrier fortifying intestinal integrity and gut functionality.

## 1. Introduction

Irritable bowel syndrome (IBS), also known as spastic colon, is considered a chronic disorder affecting the gastrointestinal (GI) tract, with a worldwide prevalence of 10–15%, with women being more predisposed than men (39.5% vs. 24.9%, respectively) [1]. Its prevalence also increases proportionally with age. It has been reported that constipation linked with IBS decreases colonic mucus and mucosal thickness, leading to mucosal damage in the deep colonic layers [2]. To provide targeted treatment to patients with IBS, researchers and clinicians have used various classification criteria, such as the Rome criterion (I to IV) in which [3] the Rome IV defines IBS as a functional bowel disorder characterized by abdominal pain associated with defecation or a change in bowel habits. 

Much attention has been paid to IBS-C, defined as a chronic disease characterized by abdominal pain, cramping and bloating, associated with defecatory problems and difficulty in evacuating stools. Yet, the pathogenesis of IBS-C is still not fully understood although there is growing evidence that constipation is influenced by several environmental factors, including xenobiotics exposure, stress, and dietary habits. Moreover, intestinal changes such as dysbiosis and altered permeability of the epithelial barrier have been observed within the colon and jejunum tracts in IBS-C patients. Furthermore, it has been shown that IBS-C negatively impacts patients’ daily lives and their mental health, with depression as the most common psychological consequence of the disease [4]. Today, a variety of therapeutic options exist to manage IBS-C Tricyclic Antidepressants (TCA), anticholinergic drugs and pain-relieving drugs are capable of relieving abdominal pain by relaxing the muscle of the intestinal wall [5]. Nevertheless, their use is limited due to the possible side effects that may occur following their administration, such as nausea and vomiting, dizziness, drowsiness, and interactions with other drugs. Non-pharmacological options have gained traction over the years: several studies have indicated that certain probiotics such as Bifidobacterium and Lactobacillus as well as prebiotics [6], are able to promote regular bowel movements and health. In addition, polyphenols and polysaccharides can relieve constipation symptoms [7], however, since current solutions mainly focus on relieving symptoms rather than the cause of the disease, there is still no effective cure for the treatment of IBS-C. 

Non-pharmacological approaches continue to gain interest for being safer options while being able to counteract the progression of GI diseases. Indeed, natural compounds such as yellow tea extract [8] and short-chain fatty acids (SCFAs) have been shown to alleviate constipation by accelerating intestinal transit, increasing stool weight and restoring the altered permeability of the intestinal wall [9]. Additionally, fiber-based supplements, such as psyllium can help control constipation but is not effective in reducing abdominal pain [10]. Laxatives, such as oral magnesium hydroxide (Philips milk of magnesia) or polyethylene glycol (Miralax), also represent an alternative solution [11].

In this study, much focus has been placed on the properties of three natural compounds. Xyloglucan is a hemicellulose of vegetal origin, obtained from the seeds of the Tamarind tree (Tamarindus indica) [12]. Once taken orally, xyloglucan is stratified on the intestinal mucosa, improving its resistance, and restoring a physiological intestinal function, thanks to its muco-mimetic properties [13]. Pea protein is extracted from golden or yellow pea (Pisum sativum) from which a high content of fibers, proteins and minerals is obtained [14]. Peas are a good source of antioxidants and pea proteins may positively modulate intestinal bacteria. Their solubility, strong water- and oil-holding capacities, and emulsifying properties make them good gelling agents to be used in foods and therapeutics alike. Indeed, Xyloglucan and Pea Protein (XP) have previously been reported to restore the intestinal barrier, thanks to their muco-mimetic properties [15,16], and to improve intestinal permeability in IBS-D patients [13]. Chia seeds (CS) are small dark seeds of the chia plant, which are rich in calcium, vitamin C, omega-3 and fiber. It has been shown that chia seeds possess laxative properties, as they are considered natural bulking agents which help accelerate intestinal transit, thus managing constipation [17]. The numerous properties provided by these natural substances make them attractive alternatives to standard therapies because they are highly tolerable and can manage symptoms in addition to evoking benefits towards the integrity of the intestinal wall. Therefore, the aim of this study was to provide a deeper understanding of alternative natural approaches to restore intestinal barrier functionality, by investigating the effects of XP + CS in an in vivo model of IBS-C.

## 2. Materials and Methods

### 2.1. Materials

Tamarind seed polysaccharide (Xyloglucan), Pea Protein, and Chia seed powder were provided by DEVINTEC SAGL (Lugano, Switzerland). All compounds were weighed and diluted in saline. The doses used were the following: Xyloglucan: 100 mg; Pea protein: 175 mg; Chia Seed: 250 mg. The formulation and the duration of treatment was based to previous preclinical studies performed to assess the best ratio and efficacy respectively.

### 2.2. Animals

Adult male Sprague–Dawley rats (Harlan, Milan, Italy) were used for the experiment. Animals were housed in a controlled environment (22 ± 2 °C, 55 ± 15% relative humidity, 12 h light/dark cycle). After a one-week acclimatization, rats were fed a standard diet from Envigo (Milan, Italy) and water [18]. The animals used for this study were randomly selected from those suitable, and available at that time. The animal study was performed in accordance with Italian regulations on the use of animals (D.M.116192) and Directive legislation (EU) (2010/63/EU) amended by Regulation (EU) 2019/1010.

### 2.3. In Vivo Model of IBS-C Induction

The IBS-C induction was performed as previously reported by Xu and colleagues [19]. More specifically, the animals were fasted overnight for approximately 12 h before starting the experiment. IBS-C was induced by the gastric instillation, by oral gavage, of 2 mL of cold water (0–4 °C) while the Sham rats received 2 mL of vehicle (saline) for 14 days. The instillation time was set at 08:00 h to avoid the influence of biorhythm. 

After IBS-C induction, animals were orally treated with XP + CS for 7 days through sterilized oral gavage. The animals were sacrificed on day 22 by anesthesia and cervical dislocation. Body weight was monitored daily, from day 1 to day 22, using a standard rat-weighing machine.

### 2.4. Experimental Groups

Rats were randomly divided into the following groups:

Group 1: Sham + vehicle group, rats received an intragastric injection of saline for 14 days.
Group 2: IBS-C group, rats received gastric instillation of 2 mL of cold water for 14 days.
Group 3: IBS-C + XP + CS group, rats received gastric instillation of 2 mL of cold water for 14 days and then received XP + CS for 7 days.

### 2.5. Determination of Constipation-Related Indicators

Constipation-related indicators including the excreted feces and urine volumes of individual rats were evaluated. Food and water intake were also monitored [20]. 

### 2.6. Stool Moisture Content and Urine Volume

The rats were placed in clean and separated cages daily for approximately 6 h. For stool collection, each rat was moved into a clean cage every day and stool samples were collected throughout the experiment and used further. The freshly excreted stool was collected in sterile tubes and weighed. Subsequently, the feces were dried in an oven at 80 °C for 24 h and then reweighed [21]. The following formula was used to calculate the water content of the feces: Water content = (weight of the feces − the dried weight of the feces)/weight of the feces × 100. Urine was collected, and the volume was measured.

### 2.7. Histological Analysis

To assess intestinal changes after IBS-C induction, histological evaluation was performed as previously described [22]. Briefly, colon tissues were collected and fixed with 10% neutral formalin, embedded in paraffin, sectioned at 7 µm and stained with hematoxylin-eosin (H&E). The degree of colon histopathological change was evaluated according to a four-point scale: 0 = no change, 1 = edema formation, 2 = mild/moderate mucus layers alterations, 3 = moderate mucus layers alterations, 4 = severe intestinal alterations. Sections were observed under light microscopy (Zeiss Microscope, Axiostar Plus, Milan, Italy) at 10× magnification (100 µm). Sections were observed under light microscopy and evaluated by a histopathologist. 

Moreover, the thickness of colon parts were measured and reported in terms of μm.

### 2.8. Immunohistochemical Localization of ZO-1 and Occludin

The immunohistochemical staining was executed as previously described [23]. The following primary antibodies were used: anti-ZO-1 (#617300 Invitrogen, Carlsbad, CA, USA, 1:100 in PBS *v*/*v*), and anti-Occludin (#sc-133256; Santa Cruz Biotechnology; Dallas, TX, USA 1:100 in PBS *v*/*v*). Sections were observed under light microscopy (Zeiss Microscope, Axiostar Plus, Milan, Italy) at 10× magnification (100 µm). 

### 2.9. Visceral Sensitivity and Abdominal Pain Tests

Prior sacrifice with a subset of animals we assessed behavioral pain response during IBS-C as previously described [24].

Briefly, for abdominal response each step of inflation endured five min. Responses to applied pressure levels were measured with electromyographic recordings during the five-min interval and data are expressed as contractions/five min.

For abdominal withdrawal response (AWR) we used the method indicated by Lucarini et al. [25], through a semi-quantitative score.

AWR measurement consisted of visual observation of animal responses to graded CRD (0.5, 1, 2, 3 mL) by blinded observers who assigned AWR scores: no behavioural response to colorectal distention (0); immobile during colorectal distention and occasional head clinching at stimulus onset (1); mild contraction of the abdominal muscles but absence of abdomen lifting from the platform (2); observed strong contraction of the abdominal muscles and lifting of the abdomen off the platform (3); arching of the body and lifting of the pelvic structures and scrotum (4).

### 2.10. Statistical Analysis

All results are expressed as mean ± standard deviation (SD) of N observations, in which N represents the number of animals studied. Data are representative of at least three independent experiments. Data were analyzed with the GraphPad Prism software by One-Way and Two-Way ANOVA, followed by a post-hoc Bonferroni test for multiple comparisons. When *p*-values were less than 0.05, the results were considered statistically significant.

## 3. Results

### 3.1. Evaluation of XP + CS Treatment on Constipation-Related Factors after IBS-C Induction 

**Figure 1:**
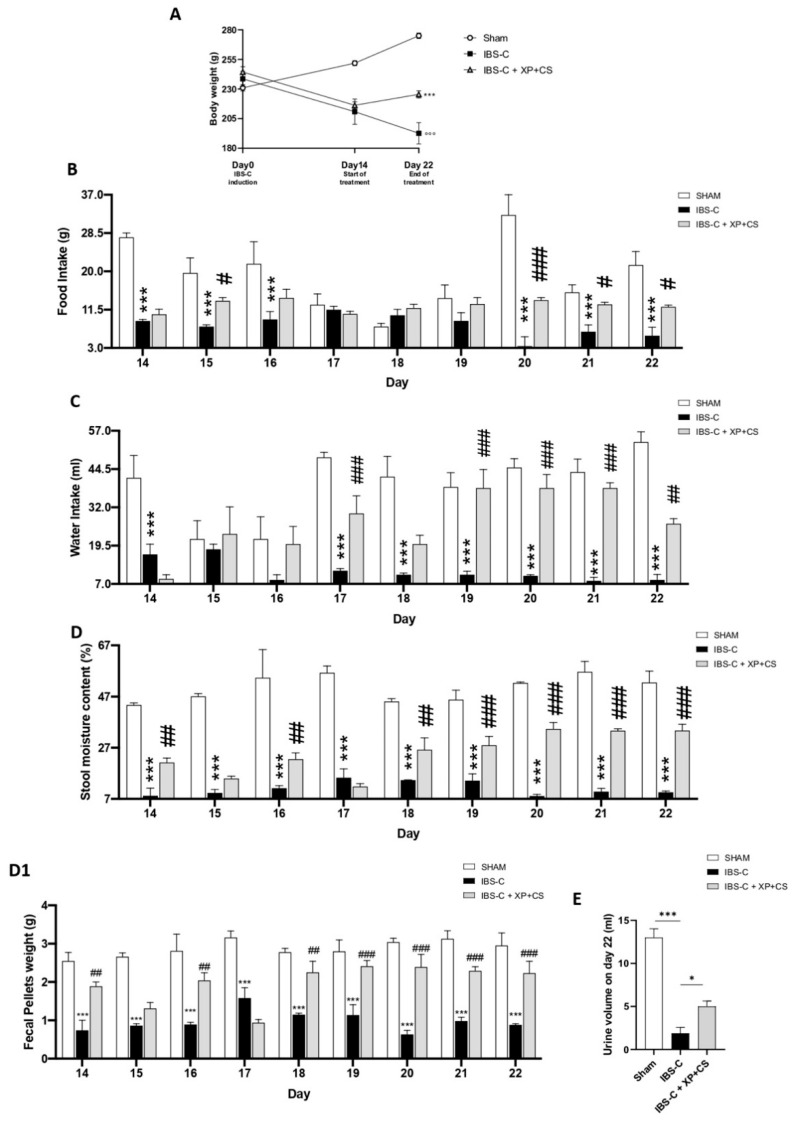
XP and CS effects on body weight, food and water intake, stool content and urine volume. (**A**) Analysis of body weight (days 0 to 22). °°° *p* < 0.001 vs. Sham; *** *p* < 0.001 vs. IBS-C. (**B**,**C**) Food and water intake measured from day 0 to 22. *** *p* < 0.001 vs. Sham; # *p* < 0.05 vs. IBS-C; ### *p* < 0.01 vs. IBS-C; ### *p* < 0.001 vs. IBS-C. (**D**) Stool moisture content percentage measured from day 0 to 22 *** *p* < 0.001 vs. Sham; ## *p* < 0.01 vs. IBS-C; ### *p* < 0.001 vs. IBS-C. (**D1**) Fecal pellets weight. *** *p* < 0.05 vs. Sham; ## *p* < 0.01 vs. IBS-C; ### *p* < 0.001 vs. IBS-C. (**E**) Urine volume measure on day 22. *** *p* < 0.001 vs. Sham; * *p* < 0.05 vs. IBS-C.

The main pathological features of IBS-C include alterations to GI motility, hard stools and painful swelling [26]. Here, the body weight of Sham rats with no constipation naturally increased from day 1 to 22. On the contrary, rats with IBS-C had significantly lower body weight compared to Sham rats, especially from day 14 to 22. However, after treatment with XP + CS for 7 days, the respective body weight was significantly increased (Figure 1 panels A). Similar trends were observed in food and water intake. The food and water intake were significantly reduced in IBS-C animals compared to Sham group. The 7-day treatment with XP + CS significantly restored food and water intake (Figure 1 panels B and C, respectively). The moisture content of the feces was used to measure the level of constipation [27]. Interestingly, IBS-C induced rats showed significantly lower stool moisture content compared to the Sham rats, whereas XP + CS treatment, given for 7 days, significantly increased the stool moisture content already within the first day of treatment and a further improvement was displayed at the end of the treatment (Figure 1 panel D). These data were also confirmed by fecal pellet weight analysis (Figure 1 Panel D1). Moreover, lower urine volume observed in IBS-C induced rats was increased by XP + CS treatment (Figure 1 panel E).

### 3.2. Efficacy of XP + CS Treatment in Resolving Colonic Damage Induced by IBS-C 

**Figure 2:**
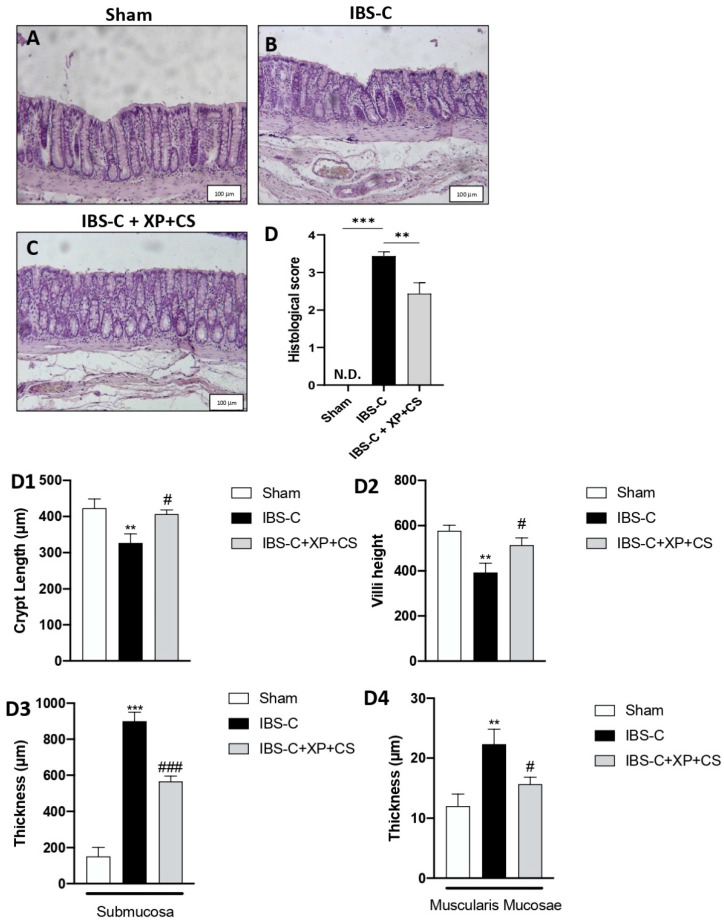
XP and CS treatment alleviate IBS constipation. Histological evaluation of colons at day 22. (**A**) Intact colon tissue from Sham group. (**B**) Colon tissue significantly damaged after IBS-C induction (IBS-C group). (**C**) XP + CS treatment alleviated colon damage after constipation (IBS-C + XP + CS group). (**D**) Histological score. (**D1**–**D4**) histomorphic parameters. *** *p* < 0.001 vs. Sham; ** *p* < 0.01 vs. Sham; ### *p* < 0.001 vs. IBS-C; # *p* < 0.005 vs. IBS-C; ND: not detectable.

The microscopic examination of colonic tissue showed that the IBS-C model induced edema formation and damage in deeper mucosal layers (muscularis, mucosa and submucosa) when compared to Sham rats (Figure 2 panels B and A, respectively, see histological score panel D). However, the colonic tissue of XP + CS treated rats showed significant amelioration in tissue architecture, reducing edema by attenuating the mucus alterations changes induced by IBS-C (Figure 1 panel C, see histological score panel E).

Significant changes were also detected in histomorphic parameters (Figure 1 panels D, D1, D3, D4).

### 3.3. XP + CS Increased Occludin and ZO-1 Expression after IBS-C Induction

**Figure 3:**
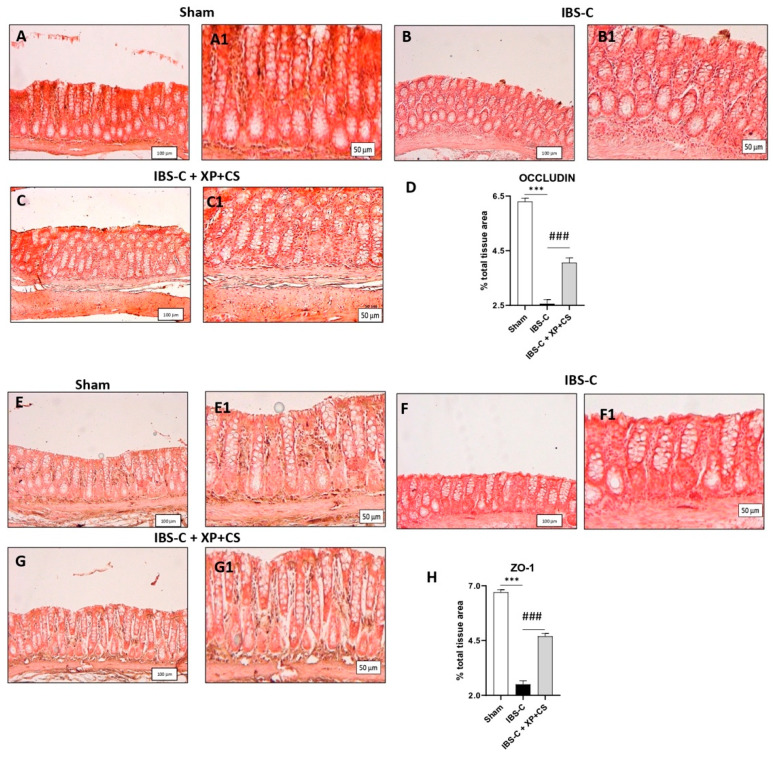
XP and CS treatment restored tight junctions’ expression after IBS constipation. (**A** and **A1**) Basal expression of Occludin in the colon of Sham rats (magnification 10x and 20x). (**B** and **B1**) Loss of tight junction Occludin immunopositivity in the colon from IBS-C rats (magnification 10x and 20x). (**C** and **C1**) XP + CS treatments restored Occludin immunopositivity after IBS-C (magnification 10x and 20x). (**D**) Percentage of Occludin signal/total area. *** *p* < 0.001 vs. Sham; *** *p* < 0.001 vs. IBS-C. (**E** and **E1**) Basal expression of ZO-1 in the colon of Sham rats (magnification 10x and 20x). (**F** and **F1**) Loss of tight junction ZO-1 immunopositivity in the colon from IBS-C rats (magnification 10x and 20x). (**G** and **G1**) XP + CS treatments restored ZO-1 immunopositivity after IBS-C (magnification 10x and 20x). (**H**) Percentage of ZO-1 signal/total area. *** *p* < 0.001 vs. Sham; ### *p* < 0.001 vs. IBS-C.

Occludin and ZO-1 represent multiprotein junctional complexes that regulate cellular permeability and barrier intestinal function; relatively it is known that their decrease leads to a disruption of colonic permeability [28]. Here, IBS-C induction in rats reduced the expression of both tight junctions proteins compared to Sham rats’ levels, (Figure 3 panels A and B, respectively for Occludin, see Occludin% of signal panel D and panels E and F respectively for ZO-1, see ZO-1% of signal panel H). Notably, the treatment with XP + CS was able to significantly restore both occludin and ZO-1 expression levels (Figure 3 panels C for occludin, see occludin% of signal panel D and panel G for ZO-1, see ZO-1% of signal panel H), improving intestinal barrier functionality.

### 3.4. XP + CS Decreased Abdominal Pain after IBS-C Induction

Abdominal visceral hypersensitivity is a feature of IBS-C patients, therefore, we examined whether XP + CS was able to counteract pain symptoms. 

**Figure 4:**
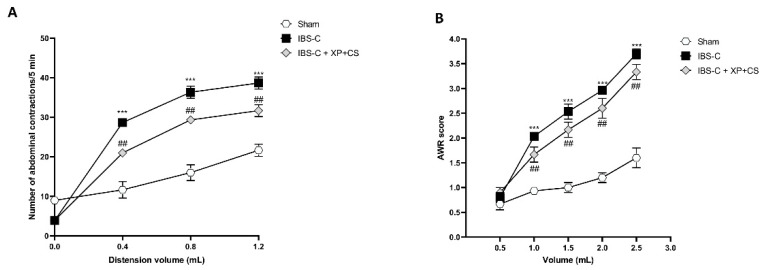
XP and CS considerably decreases abdominal pain following IBS-C. (**A**) Number of abdominal contractions measured in 5 min in Sham, IBS-C and IBS-C + XP + CS groups; (**B**) AWR to colon rectal distension (0.5, 1, 2, 3 mL balloon inflation) in Sham, IBS-C and IBS-C + XP + CS groups. *** *p* < 0.001 vs. Sham; ## *p* < 0.01 vs. IBS-C.

The data obtained highlighted that IBS-C induction increased the number of abdominal contractions and AWR compared to the Sham Group (Figure 4A,B, respectively). However, the XP + CS treatment significantly decreased IBS-C visceral hypersensitivity saline (Figure 4 panels A and B, respectively). 

## 4. Discussion

Constipation is a very complex pathological disorder characterized by altered intestinal motility [29], reduced bowel movement frequency, dry stool, and difficult defecation affecting the patient’s life quality. Not to mention, chronic and functional constipation are very difficult to distinguish between each other due to the similarity of the symptoms, such as impaired colon motility and pelvic floor muscle dysfunction; this further complicates diagnosis and treatment regimens [30]. In the last decade, a lot of attention has shifted toward understanding the role of the gut barrier. Intestinal permeability plays a crucial role in the functionality of the intestinal barrier that physiologically regulates the uptake and circulation of minerals and nutrients [31]. In IBD, for instance, an impaired permeability results in loss of intestinal homeostasis [32]. 

In the present study, the combination of xyloglucan, pea protein and chia seed powder strongly ameliorated the pathophysiological features of IBS-C. 

Xyloglucan displays various applications in medicine and nutrition by exerting protective barrier properties on the intestinal mucosa inhibiting colitis-induced intestinal alterations [33]. Particularly, the “mucosal protector” activity is due to the protective film formed in the intestinal, which exercise a mechanical effect against harmful gut bacteria. Thus, Xyloglucan is a valuable candidate to restore physiological functions of the intestinal wall, improving the mucosal resistance to pathological invaders, decreasing the gut permeability of the intestinal mucosa and avoids the adherence and proliferation of pathogens [34].

Pea protein, a widely used ingredient in the food industry, has been shown to create a synergistic mechanical film that can improve symptoms of acute diarrhea and abdominal pain [35]. Pea protein exert an emollient and soothing action in the digestive tract thanks to their high fiber content while modulating intestinal bacteria activities, overall resulting in a remarkable improvement of gut mucosal barrier and gut homeostasis [18].

Moreover, dietary fibers can exert a laxative action if taken with plenty of water, as they are able to increase the volume of feces [36]. 

Similarly, CS are very rich in fiber and can be a valid aid for the regularity of the intestine. This beneficial activity is attributable to CS mucilage, which give intestinal relief and fecal regularization by modulating intestinal bacterial microflora [37]. 

Here, the combined action of muco-mimetic substances and a laxative were capable of restoring gut barrier integrity and regulating intestinal motility. Indeed, XP + CS administered daily for 7 days improved intestinal motility facilitating stool transit through the intestine. In addition, XP + CS treatment ameliorated constipation by influencing constipation-related factors such as food and water intake, thus counteracting weight loss. These results suggest that XP + CS treatment for 7 days promotes intestinal peristalsis, ameliorating the prolonged and disabling intestinal alteration due to constipation as well as abdominal pain. 

We observed that XP + CS treatment was able to restore intestinal mucosal architecture in constipated rats, by creating a protective barrier over the damaged epithelial cells. In IBS associated with constipation, the loss of intestinal functional integrity facilitates external agents’ to penetrate the intestinal mucosa further increasing paracellular epithelial permeability [38]. In this study, we observed a compromised expression of tight junctions-related proteins such as ZO-1 and Occludin in the colonic tissue of constipated rats which were significantly lower compared to the Sham group. Interestingly, XP + CS treatment restored tight junctions’ levels indicating that the synergistic mechanical barrier was able to prevent epithelial damage in the colonic tissue of constipated rats. Therefore, the results obtained in this study demonstrate that the administration of XP + CS could represent a promising alternative for patients suffering from IBS-C. However, this study faces limitations related to the fact that the murine model of IBS-C is considered a reliable model, but not identical to the human condition. Other studies including a clinical investigation are needed to better describe the mechanism of action and beneficial properties these natural compounds may provide to IBS-C patients. Moreover, microbiota composition which could be influenced by IBS onset needs to be investigated in addition to evaluating GI motor abnormalities or nutrient absorption scores. 

## 5. Conclusions

These results demonstrate that XP + CS treatment promotes the recovery of constipation-related parameters by influencing GI transit and motility, and histopathology alterations in rats with IBS-C. Our results further suggest that the combined effects of these compounds are associated with an improvement of intestinal barrier integrity with the ability to restore tight junctions’ expression. Taken together, our findings indicate that XP + CS treatment could be considered a potential candidate for the treatment of IBS-C and functional constipation thanks to their safety profile and unique mechanism of action.

## Data Availability

Not applicable.

## References

[1] Ardizzone A., Filippone A., Mannino D., Scuderi S.A., Casili G., Lanza M., Cucinotta L., Campolo M., Esposito E. (2022). Ulva pertusa, a Marine Green Alga, Attenuates DNBS-Induced Colitis Damage via NF-kappaB/Nrf2/SIRT1 Signaling Pathways. J. Clin. Med..

[2] Li C., Nie S.P., Zhu K.X., Xiong T., Li C., Gong J., Xie M.Y. (2015). Effect of Lactobacillus plantarum NCU116 on loperamide-induced constipation in mice. Int. J. Food Sci. Nutr..

[3] Lacy B.E., Patel N.K. (2017). Rome Criteria and a Diagnostic Approach to Irritable Bowel Syndrome. J. Clin. Med..

[4] Kopczynska M., Mokros L., Pietras T., Malecka-Panas E. (2018). Quality of life and depression in patients with irritable bowel syndrome. Przegląd Gastroenterol..

[5] Gillman P.K. (2007). Tricyclic antidepressant pharmacology and therapeutic drug interactions updated. Br. J. Pharmacol..

[6] Marlicz W., Skonieczna-Zydecka K., Krynicka P., Loniewski I., Rydzewska G. (2021). Probiotics in irritable bowel syndrome—Is the quest for the right strain over? Rapid review of existing guidelines and recommendations. Przegląd Gastroenterol..

[7] Roudsari N.M., Lashgari N.A., Momtaz S., Farzaei M.H., Marques A.M., Abdolghaffari A.H. (2019). Natural polyphenols for the prevention of irritable bowel syndrome: Molecular mechanisms and targets; a comprehensive review. Daru.

[8] Cao P.Q., Li X.P., Ou-Yang J., Jiang R.G., Huang F.F., Wen B.B., Zhang X.N., Huang J.A., Liu Z.H. (2021). The protective effects of yellow tea extract against loperamide-induced constipation in mice. Food Funct..

[9] Lanza M., Filippone A., Ardizzone A., Casili G., Paterniti I., Esposito E., Campolo M. (2021). SCFA Treatment Alleviates Pathological Signs of Migraine and Related Intestinal Alterations in a Mouse Model of NTG-Induced Migraine. Cells.

[10] Soltanian N., Janghorbani M. (2019). Effect of flaxseed or psyllium vs. placebo on management of constipation, weight, glycemia, and lipids: A randomized trial in constipated patients with type 2 diabetes. Clin. Nutr. ESPEN.

[11] Oh S.J., Fuller G., Patel D., Khalil C., Spalding W., Nag A., Spiegel B.M.R., Almario C.V. (2020). Chronic Constipation in the United States: Results From a Population-Based Survey Assessing Healthcare Seeking and Use of Pharmacotherapy. Am. J. Gastroenterol..

[12] Campolo M., Casili G., Paterniti I., Filippone A., Lanza M., Ardizzone A., Scuderi S.A., Cuzzocrea S., Esposito E. (2020). Effect of a Product Containing Xyloglucan and Pea Protein on a Murine Model of Atopic Dermatitis. Int. J. Mol. Sci..

[13] de Los Rios C.C., Falcon B.S., Arguelles-Arias F., Perez E., Teruel C., Geijo F., Rey E. (2021). Long-term safety and efficacy study of a medical device containing xyloglucan, pea protein reticulated with tannins and xylo-oligosaccharides, in patients with diarrhoea-predominant irritable bowel syndrome. Ther. Adv. Gastroenterol..

[14] Emkani M., Oliete B., Saurel R. (2021). Pea Protein Extraction Assisted by Lactic Fermentation: Impact on Protein Profile and Thermal Properties. Foods.

[15] Bellini M., Berti G., Bonfrate L., Ciranni F., Di Ciaula A., Di Ruscio M., Dell’Era A., Lambiase C., Noto A., Pancetti A. (2021). Use of GELSECTAN((R)) in Patients with Irritable Bowel Syndrome (IBS): An Italian Experience. Patient Prefer. Adherence.

[16] Garland V., Herlitz L., Regunathan-Shenk R. (2020). Diet-induced oxalate nephropathy from excessive nut and seed consumption. BMJ Case Rep..

[17] Kulczynski B., Kobus-Cisowska J., Taczanowski M., Kmiecik D., Gramza-Michalowska A. (2019). The Chemical Composition and Nutritional Value of Chia Seeds-Current State of Knowledge. Nutrients.

[18] Ardizzone A., Lanza M., Casili G., Campolo M., Paterniti I., Cuzzocrea S., Esposito E. (2022). Efficacy of a Novel Therapeutic, Based on Natural Ingredients and Probiotics, in a Murine Model of Multiple Food Intolerance and Maldigestion. Nutrients.

[19] Xu J.R., Luo J.Y., Shang L., Kong W.M. (2006). Effect of change in an inhibitory neurotransmitter of the myenteric plexus on the pathogenetic mechanism of irritable bowel syndrome subgroups in rat models. Chin. J. Dig. Dis..

[20] Lee H.Y., Kim J.H., Jeung H.W., Lee C.U., Kim D.S., Li B., Lee G.H., Sung M.S., Ha K.C., Back H.I. (2012). Effects of Ficus carica paste on loperamide-induced constipation in rats. Food Chem. Toxicol..

[21] Xie N., Cui Y., Yin Y.N., Zhao X., Yang J.W., Wang Z.G., Fu N., Tang Y., Wang X.H., Liu X.W. (2011). Effects of two Lactobacillus strains on lipid metabolism and intestinal microflora in rats fed a high-cholesterol diet. BMC Complement. Altern. Med..

[22] Campolo M., Crupi R., Cordaro M., Cardali S.M., Ardizzone A., Casili G., Scuderi S.A., Siracusa R., Esposito E., Conti A. (2021). Co-Ultra PEALut Enhances Endogenous Repair Response Following Moderate Traumatic Brain Injury. Int. J. Mol. Sci..

[23] Filippone A., Casili G., Ardizzone A., Lanza M., Mannino D., Paterniti I., Esposito E., Campolo M. (2021). Inhibition of Prolyl Oligopeptidase Prevents Consequences of Reperfusion following Intestinal Ischemia. Biomedicines.

[24] Scuderi S.A., Casili G., Lanza M., Ardizzone A., Pantaleo L., Campolo M., Paterniti I., Cucinotta L., Cuzzocrea S., Esposito E. (2022). Efficacy of a Product Containing Xyloglucan and Pea Protein on Intestinal Barrier Function in a Partial Restraint Stress Animal Model. Int. J. Mol. Sci..

[25] Lucarini E., Nocentini A., Bonardi A., Chiaramonte N., Parisio C., Micheli L., Toti A., Ferrara V., Carrino D., Pacini A. (2021). Carbonic Anhydrase IV Selective Inhibitors Counteract the Development of Colitis-Associated Visceral Pain in Rats. Cells.

[26] Saha L. (2014). Irritable bowel syndrome: Pathogenesis, diagnosis, treatment, and evidence-based medicine. World J. Gastroenterol..

[27] Qian Y., Suo H., Du M., Zhao X., Li J., Li G.J., Song J.L., Liu Z. (2015). Preventive effect of Lactobacillus fermentum Lee on activated carbon-induced constipation in mice. Exp. Ther. Med..

[28] Meng J., Yu H., Ma J., Wang J., Banerjee S., Charboneau R., Barke R.A., Roy S. (2013). Morphine induces bacterial translocation in mice by compromising intestinal barrier function in a TLR-dependent manner. PLoS ONE.

[29] Sharma A., Rao S. (2017). Constipation: Pathophysiology and Current Therapeutic Approaches. Handb. Exp. Pharmacol..

[30] Vriesman M.H., Koppen I.J.N., Camilleri M., Di Lorenzo C., Benninga M.A. (2020). Management of functional constipation in children and adults. Nat. Rev. Gastroenterol. Hepatol..

[31] Hossen I., Hua W., Ting L., Mehmood A., Jingyi S., Duoxia X., Yanping C., Hongqing W., Zhipeng G., Kaiqi Z. (2020). Phytochemicals and inflammatory bowel disease: A review. Crit. Rev. Food Sci. Nutr..

[32] Hagan M., Hayee B.H., Rodriguez-Mateos A. (2021). (Poly)phenols in Inflammatory Bowel Disease and Irritable Bowel Syndrome: A Review. Molecules.

[33] Pique N., Gomez-Guillen M.D.C., Montero M.P. (2018). Xyloglucan, a Plant Polymer with Barrier Protective Properties over the Mucous Membranes: An Overview. Int. J. Mol. Sci..

[34] Campolo M., Lanza M., Filippone A., Paterniti I., Casili G., Scuderi S.A., Ardizzone A., Cuzzocrea S., Esposito E. (2020). Evaluation of a Product Containing Xyloglucan and Pea Protein on Skin Barrier Permeability. Skin Pharmacol. Physiol..

[35] Gnessi L., Bacarea V., Marusteri M., Pique N. (2015). Xyloglucan for the treatment of acute diarrhea: Results of a randomized, controlled, open-label, parallel group, multicentre, national clinical trial. BMC Gastroenterol..

[36] Cong L., Ma J.T., Jin Z.J., Duan L.W., Su W.P., Zheng J., Zhang L.J., Xu J., Li D.F. (2015). Efficacy and Safety of High Specific Volume Polysaccharide-A New Type of Dietary Fiber for Treatment of Functional Constipation and IBS-C. J. Nutr. Sci. Vitaminol. (Tokyo).

[37] Tamargo A., Cueva C., Laguna L., Moreno-Arribas M.V., Muñoz L.A. (2018). Understanding the impact of chia seed mucilage on human gut microbiota by using the dynamic gastrointestinal model simgi^®^. J. Funct. Foods.

[38] Turner J.R. (2009). Intestinal mucosal barrier function in health and disease. Nat. Rev. Immunol..

